# Identification of candidate drugs using tensor-decomposition-based unsupervised feature extraction in integrated analysis of gene expression between diseases and DrugMatrix datasets

**DOI:** 10.1038/s41598-017-13003-0

**Published:** 2017-10-23

**Authors:** Y.-h. Taguchi

**Affiliations:** Department of Physics, Chuo University, 1-13-27 Kasuga, Bunkyo-ku, Tokyo, 112-8551 Japan

## Abstract

Identifying drug target genes in gene expression profiles is not straightforward. Because a drug targets proteins and not mRNAs, the mRNA expression of drug target genes is not always altered. In addition, the interaction between a drug and protein can be context dependent; this means that simple drug incubation experiments on cell lines do not always reflect the real situation during active disease. In this paper, I applied tensor-decomposition-based unsupervised feature extraction to the integrated analysis using a mathematical product of gene expression in various diseases and gene expression in the DrugMatrix dataset, where comprehensive data on gene expression during various drug treatments of rats are reported. I found that this strategy, in a fully unsupervised manner, enables researchers to identify a combined set of genes and compounds that significantly overlap with gene and drug interactions identified in the past. As an example illustrating the usefulness of this strategy in drug discovery experiments, I considered cirrhosis, for which no effective drugs have ever been proposed. The present strategy identified two promising therapeutic-target genes, CYPOR and HNFA4; for their protein products, bezafibrate was identified as a promising candidate drug, supported by *in silico* docking analysis.

## Introduction

*In silico* drug discovery is an important task because experimental identification and verification of therapeutic compounds are time-consuming and expensive processes. There are two major trends in *in silico* drug discovery: the ligand-based approach^1^ and structure-based approach^2^. The former is straightforward: new drug candidates are identified based upon the similarity with known drugs no matter how the similarity is evaluated. Although it is a powerful method, there are some drawbacks: if there are no known drugs for target proteins, then there is no way to find new drug candidates. Even if there are many known drugs for the target protein, it is hopeless to try to find compounds that are effective but without any similarity to known drugs. The second, structure-based approach, can address these weaknesses. It can identify new therapeutic compounds even without the information about known drugs. Of course, there are some drawbacks in this strategy too. If the target protein’s structure is not known, it must be predicted prior to the drug discovery process. Even if the target protein’s structure is known, because we need to numerically verify the binding affinity between the ligand compound and target protein – this work also requires a large amount of computational resources – structure-based *in silico* drug discovery is still far from easy to implement. In addition, the accuracy of prediction of protein structure and of ligand-binding structure is not very high^3^. Thus, it would be helpful to have an additional or alternative strategy for *in silico* drug discovery.

Recently, an alternative approach was proposed that is aimed at finding drug candidates computationally using gene expression profiles of cell lines treated with compounds^4,5^. This third approach is not straightforward. Firstly, because compounds target not mRNAs but proteins, mRNA expression of drug target proteins is not always affected. Therefore, direct identification of a drug target protein in gene expression data cannot be done. Secondly, gene expression alteration caused by treatment with a compound may be context dependent; in other words, in a cell line, the gene expression change caused by incubation with a compound may differ from that in diseases. To compensate for these difficulties, the gene expression signature strategy was developed^6,7^. In this approach, gene expression alteration profiles caused by treatment of a cell line with various drug candidates are compared with those of known drugs. If the profiles are similar, then new drug candidates are expected to function similarly to known drugs. Although this third strategy is useful, if there are no known drugs for the disease under study, then this approach cannot function, as in the case of ligand-based methods. In this paper, I propose a strategy that can infer drug candidates from profiles of drug treatment-associated gene expression without the information about known compounds for diseases. In this strategy, I employ the tensor decomposition (TD)-based unsupervised feature extraction (FE) approach, which is an extension of the recently proposed principal component analysis (PCA)-based unsupervised FE; PCA-based unsupervised FE has successfully solved various bioinformatic problems^8–28^. PCA-based unsupervised FE has also been often applied to integrated analysis. In their integrated approach, after PCA-based unsupervised FE is applied to an individual dataset separately, genes are selected from each of these datasets. Then, intersections among them are evaluated as gene sets carrying greater confidence. This strategy has been applied to the identification of genes associated with aberrant promoter methylation common among three autoimmune diseases^14^, identification of genes commonly affected by two histone deacetylase inhibitors^11^, and identification of reliable biomarkers considering microRNA, mRNA expression, and compounds together^20^. The advantage of these integrated analyses using PCA-based unsupervised FE compared with standard integrated analysis is that there is no need for a weight factor, which is necessary to integrate multiple datasets because PCA-based unsupervised FE was applied to an individual dataset separately. On the other hand, this strategy, i.e. integrated analysis by applying PCA-based unsupervised FE to an individual dataset separately also has some drawbacks: when there are no common gene sets, there is no way to proceed with the analysis although there are rarely no common gene sets. To compensate for this weakness without introducing weights, in the newly developed TD-based strategy here, tensors are generated using a mathematical product of a gene expression profile of drug-treated cell lines and of a gene expression profile of a disease. This approach does not require any weights that a linear combination inevitably requires. Then, pairs of compounds and genes are identified, where the gene’s mRNA expression alteration is associated with drug-treated cell lines and is simultaneously coincident with such an alteration during disease progression. Drug target genes are further inferred based upon gene expression profiles when single-gene knockout or overexpression experiments are conducted. In this study, the inferred gene–compound interactions were found to significantly overlap with known gene–compound interactions.

## Results

### TD-based unsupervised FE is applied to a combined tensor

**Figure 1:**
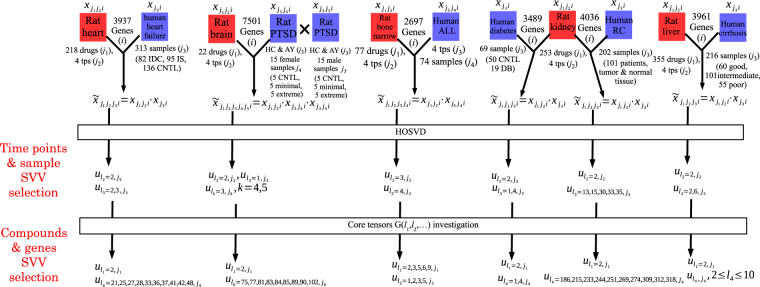
Schematics that illustrate the procedure of TD-based unsupervised FE applied to the various disease and DrugMatrix datasets. SVV: singular value vector.

In this study, to demonstrate the usefulness of the present strategy even when applied to various diseases, I consider application to multiple diseases as follows. In the text that follows, I describe detailed results for all the diseases analysed in this study. For a summary of this section, see Table 1 and Fig. 1

**Table 1:** A summary of TDs and identification of various singular value vectors for identification of candidate drugs and genes used to find genes encoding drug target proteins.

diseases	tensors			core tensor	singular value vectors
	DrugMatrix	disease	generated		
heart failure	${\tilde{x}}_{{j}_{1}{j}_{2}i}\in {{\mathbb{R}}}^{{N}_{1}\times {N}_{2}\times {N}_{4}}$	${x}_{{j}_{3}i}\in {{\mathbb{R}}}^{{N}_{3}\times {N}_{4}}$	${x}_{{j}_{1}{j}_{2}{j}_{3}i}$	$G({\ell }_{1}{\ell }_{2}{\ell }_{3}{\ell }_{4})$	${u}_{{\ell }_{k},{j}_{k}},k\le 3,{u}_{{\ell }_{4},i}\in {{\mathbb{R}}}^{{N}_{k}\times {N}_{k}}$ $({N}_{1},{N}_{2},{N}_{3},{N}_{4})=(218,4,313,3937)$
			$$\in {{\mathbb{R}}}^{{N}_{1}\times {N}_{2}\times {N}_{3}\times {N}_{4}}$$		
Selected				${\ell }_{1}\mathrm{=2;}{\ell }_{2}\mathrm{=2;}{\ell }_{3}\mathrm{=2,3;}{\ell }_{4}\mathrm{=21,25,27,28,33,36,37,41,42,48}$	
PTSD rat model	${\tilde{x}}_{{j}_{1}{j}_{2}i}\in {{\mathbb{R}}}^{{N}_{1}\times {N}_{2}\times {N}_{6}}$	${x}_{{j}_{3}{j}_{k}i},k\mathrm{=4,5}\in {{\mathbb{R}}}^{{N}_{3}\times {N}_{k}\times {N}_{6}}$	${x}_{{j}_{1}{j}_{2}{j}_{3}{j}_{4}{j}_{5}i}$	$G({\ell }_{1}{\ell }_{2}{\ell }_{3}{\ell }_{4}{\ell }_{5}{\ell }_{6})$	${u}_{{\ell }_{k},{j}_{k}},k\le 5,{u}_{{\ell }_{6},i}\in {{\mathbb{R}}}^{{N}_{k}\times {N}_{k}}$ $({N}_{1},{N}_{2},{N}_{3},{N}_{4},{N}_{5},{N}_{6})=(22,4,2,15,15,7501)$
			$$\in {{\mathbb{R}}}^{{N}_{1}\times {N}_{2}\times {N}_{3}\times {N}_{4}\times {N}_{5}\times {N}_{6}}$$		
Selected				${\ell }_{1}\mathrm{=2;}{\ell }_{2}\mathrm{=2;}{\ell }_{3}\mathrm{=1;}{\ell }_{4}={\ell }_{5}\mathrm{=3;}{\ell }_{6}\mathrm{=75,77,81,83,84,85,89,90,102}$	
ALL	${\tilde{x}}_{{j}_{1}{j}_{2}i}\in {{\mathbb{R}}}^{{N}_{1}\times {N}_{2}\times {N}_{5}}$	${x}_{{j}_{3}{j}_{4}i}\in {{\mathbb{R}}}^{{N}_{3}\times {N}_{4}\times {N}_{5}}$	${x}_{{j}_{1}{j}_{2}{j}_{3}{j}_{4}i}$	$G({\ell }_{1}{\ell }_{2}{\ell }_{3}{\ell }_{4}{\ell }_{5})$	${u}_{{\ell }_{k},{j}_{k}},k\le 4,{u}_{{\ell }_{5},i}\in {{\mathbb{R}}}^{{N}_{k}\times {N}_{k}}$ $({N}_{1},{N}_{2},{N}_{3},{N}_{4},{N}_{5})=(77,4,4,74,2597)$
			$$\in {{\mathbb{R}}}^{{N}_{1}\times {N}_{2}\times {N}_{3}\times {N}_{4}\times {N}_{5}}$$		
Selected				${\ell }_{1}\mathrm{=2,3,5,6,9,10;}{\ell }_{2}\mathrm{=3;}{\ell }_{3}\mathrm{=4;}{\ell }_{5}\mathrm{=1,2,3,5}$	
diabetes	${\tilde{x}}_{{j}_{1}{j}_{2}i}\in {{\mathbb{R}}}^{{N}_{1}\times {N}_{2}\times {N}_{4}}$	${x}_{{j}_{3}i}\in {{\mathbb{R}}}^{{N}_{3}\times {N}_{4}}$	${x}_{{j}_{1}{j}_{2}{j}_{3}i}$	$G({\ell }_{1}{\ell }_{2}{\ell }_{3}{\ell }_{4})$	${u}_{{\ell }_{k},{j}_{k}},k\le \mathrm{3,}{u}_{{\ell }_{4},i}\in {{\mathbb{R}}}^{{N}_{k}\times {N}_{k}}$ $({N}_{1},{N}_{2},{N}_{3},{N}_{4})=(253,4,69,3489)$
			$$\in {{\mathbb{R}}}^{{N}_{1}\times {N}_{2}\times {N}_{3}\times {N}_{4}}$$		
Selected				${\ell }_{1}\mathrm{=2;}{\ell }_{2}\mathrm{=2;}{\ell }_{3}\mathrm{=1,4;}{\ell }_{4}\mathrm{=1,4}$	
renal carcinoma	${\tilde{x}}_{{j}_{1}{j}_{2}i}\in {{\mathbb{R}}}^{{N}_{1}\times {N}_{2}\times {N}_{4}}$	${x}_{{j}_{3}i}\in {{\mathbb{R}}}^{{N}_{3}\times {N}_{4}}$	${x}_{{j}_{1}{j}_{2}{j}_{3}i}$	$G({\ell }_{1}{\ell }_{2}{\ell }_{3}{\ell }_{4})$	${u}_{{\ell }_{k},{j}_{k}},k\le 3,{u}_{{\ell }_{4},i}\in {{\mathbb{R}}}^{{N}_{k}\times {N}_{k}}$ $({N}_{1},{N}_{2},{N}_{3},{N}_{4})=(253,4,202,4036)$
			$$\in {{\mathbb{R}}}^{{N}_{1}\times {N}_{2}\times {N}_{3}\times {N}_{4}}$$		
Selected				${\ell }_{1}\mathrm{=2;}{\ell }_{2}\mathrm{=2;}{\ell }_{3}\mathrm{=13,15,30,33,35;}{\ell }_{4}\mathrm{=186,215,233,244,251,269,274,309,312,318}$	
cirrhosis	${\tilde{x}}_{{j}_{1}{j}_{2}i}\in {{\mathbb{R}}}^{{N}_{1}\times {N}_{2}\times {N}_{4}}$	${x}_{{j}_{3}i}\in {{\mathbb{R}}}^{{N}_{3}\times {N}_{4}}$	${x}_{{j}_{1}{j}_{2}{j}_{3}i}$	$G({\ell }_{1}{\ell }_{2}{\ell }_{3}{\ell }_{4})$	${u}_{{\ell }_{k},{j}_{k}},k\le 3,{u}_{{\ell }_{4},i}\in {{\mathbb{R}}}^{{N}_{k}\times {N}_{k}}$ $({N}_{1},{N}_{2},{N}_{3},{N}_{4})=(355,4,216,3961)$
			$$\in {{\mathbb{R}}}^{{N}_{1}\times {N}_{2}\times {N}_{3}\times {N}_{4}}$$		
Selected				${\ell }_{1}\mathrm{=2;}{\ell }_{2}\mathrm{=2;}{\ell }_{3}\mathrm{=2,6;2}\le {\ell }_{4}\le 10$	

In all cases, ${\ell }_{1}$ stands for singular value vectors of compounds, whereas ${\ell }_{k}$ with the last (largest) *k* denotes gene singular value vectors. *k* stands for singular value vectors of time points in DrugMatrix data. The remaining singular value vectors correspond to sample singular value vectors dependent on the properties of gene expression profiles of diseases. See also Fig. 1 for the corresponding data.

.

### Combined analysis of human diseases and rat tissues

Here, I focused on multiple human diseases and rat tissues for the following reason. If the proposed strategy is tested on only a single combination of human diseases and rat tissues, then the probability of success is rather low. Therefore, it is better to test the strategy on as many combinations as possible. On the other hand, if the number of compounds tested on some rat tissue is not large enough, then this tissue is not suitable for the present study because the purpose of this study is to apply TD-based unsupervised FE to drug discovery; a smaller number of tested compounds means fewer opportunities for identification of an effective drug. Using this criterion, I selected five tissues, i.e. heart, brain, born narrow, kidney, and liver tissues, on which 22–315 drugs have been tested.

Next, I selected diseases with which the five tissues are analysed together. During this selection, diseases that are supposed to take place in the corresponding tissues should be chosen; otherwise, correct drug selection is unlikely. For heart tissue, I used heart failure. For brain tissue, I used post-traumatic stress disorder (PTSD). For bone narrow tissue, I selected acute lymphocytic leukaemia (ALL). For kidney tissue, I used diabetes and cancer. For liver tissue, I chose cirrhosis. The reason why I used two diseases with kidney tissue is to check whether distinct genes are identified for different diseases when I use distinct gene expression profiles.

### Heart failure

In gene expression profiles of the rat left ventricle (LV) treated with 218 drugs, I selected four time points (1/4, 1, 3, and 5 days after treatment); this is because there were substantially smaller numbers of compounds tested for other time points than for these four. On the other hand, human heart gene expression profiles represent 82 patients with idiopathic dilated cardiomyopathy, 95 patients with ischemic stroke, and 136 healthy controls. In these profiles, 3937 genes sharing gene symbols between humans and rats were considered. Then the generated tensor is

1 $${\tilde{x}}_{{j}_{1}{j}_{2}{j}_{3}i}={x}_{{j}_{1}{j}_{2}i}\cdot {x}_{{j}_{3}i},$$

where ${\tilde{x}}_{{j}_{1}{j}_{2}{j}_{3}i}\in {{\mathbb{R}}}^{218\times 4\times 313\times 3937}$, which represents a mathematical product of gene expression of the *i* th gene of LV treated with the *j*_1_ th compound at the *j*_2_ th time point after the drug treatment, ${x}_{{j}_{1}{j}_{2}i}\in {{\mathbb{R}}}^{218\times 4\times 3937}$, and gene expression of the *j*_3_ th human heart, ${x}_{{j}_{3}i}\in {{\mathbb{R}}}^{313\times 3937}$, respectively. Higher-order singular value decomposition (HOSVD) was applied to ${\tilde{x}}_{{j}_{1}{j}_{2}{j}_{3}i}$, and then core tensor $G({\ell }_{1}{\ell }_{2}{\ell }_{3}{\ell }_{4})\in {{\mathbb{R}}}^{218\times 4\times 313\times 3937}$, compound singular value matrix ${u}_{{\ell }_{1}{j}_{1}}\in {{\mathbb{R}}}^{218\times 218}$, time point singular value matrix ${u}_{{\ell }_{2}{j}_{2}}\in {{\mathbb{R}}}^{4\times 4}$, human sample singular value matrix ${u}_{{\ell }_{3}{j}_{3}}\in {{\mathbb{R}}}^{313\times 313}$, and gene singular value matrix ${u}_{{\ell }_{4}i}\in {{\mathbb{R}}}^{3937\times 3937}$ were obtained.

**Figure 2:**
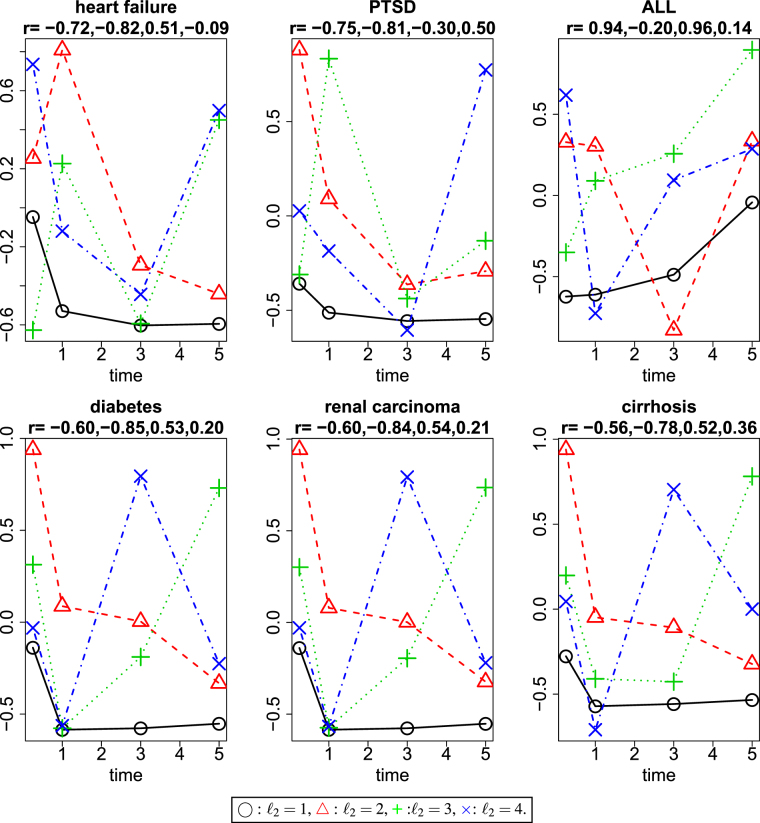
Time point singular value vectors. *r* represents Pearson’s correlation coefficients between time points (1/4, 1, 3, and 5 days after a treatment) and the first to forth singular value vectors of time points, ${x}_{{\ell }_{2},{j}_{2}}\mathrm{,1}\le {j}_{2},{\ell }_{2}\le 4$. Black open circles: ${\ell }_{2}=1$, red open triangles: ${\ell }_{2}=2$, green crosses: ${\ell }_{2}=3$, and blue crosses: ${\ell }_{2}=4$. *j*_2_ = 1, 2, 3, 4 correspond to time points 1/4, 1, 3, and 5 days, respectively.

Prior to selection of genes and compounds, we need to know which singular value vector of time points, ${u}_{{\ell }_{2}{j}_{2}}$, represents time dependence (Fig. 2). Then, I decided to use the second singular value vectors (${\ell }_{2}=2$) for heart failure.

We also need to know which sample singular value vectors generated from gene expression of disease samples are associated with the difference between healthy controls and diseases. These data must be evaluated on a case-to-case basis because gene expression profiles of disease samples do not have any unified format.

**Figure 3:**
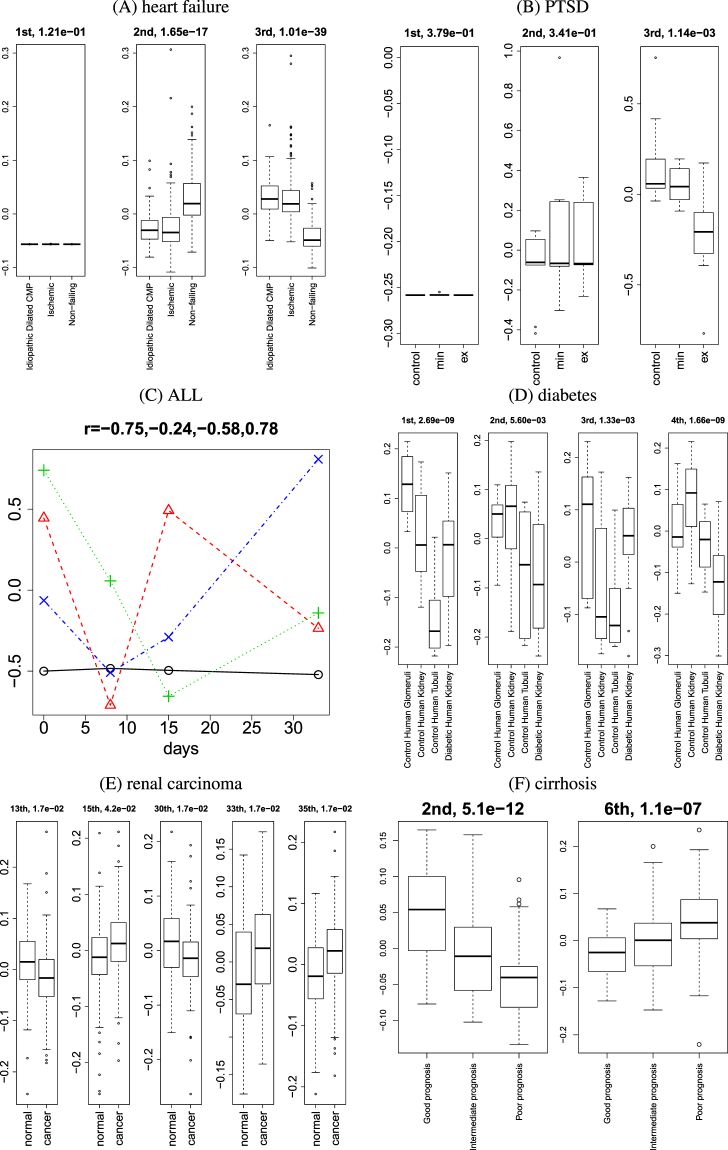
Boxplots [for (**A**), (**B**), (**D**), (**E**), and (**F**)] and time dependence (**C**) for sample singular value vectors. The numbers above boxplots are *P*-values for (**A**), (**B**), and (**D**) and adjusted *P*-vales for (**E**) and (**F**), computed by categorical regression (in other words, ANOVA). The numbers above time dependence (**C**) are correlation coefficients. (**C**) Black open circles: ${\ell }_{3}=1$, red open triangles: ${\ell }_{3}=2$, green crosses: ${\ell }_{3}=3$, and blue crosses: ${\ell }_{3}=4$. *j*_3_ = 1, 2, 3, 4 correspond to time points 0, 8, 15, and 33 days, respectively.

As for heart failure, samples are composed of two disease classes and controls. Because it is obvious that the second and third singular value vectors of samples, ${u}_{{\ell }_{3}\in \mathrm{(2,3),}{j}_{3}}$, show a difference between patients and healthy controls (Fig. 3A), I decided to employ these two for the selection of singular value vectors for identification of genes and drugs in the analysis below. The associated *P*-values are also small enough even after Benjamini–Hochberg (BH) criterion corrections.

**Figure 4:**
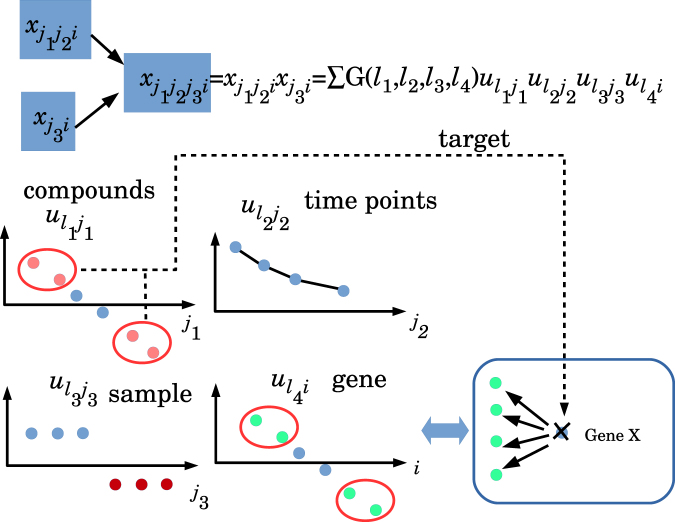
Intuitive illustration of the present strategy. Suppose there is a tensor, *x*_*j*1*j*2*i*_, which represents the *i* th gene expression at the *j*_2_ th time point after the *j*_1_ th compound is given to a rat; these data are taken from the DrugMatrix^44^ dataset. There is also a matrix, *x*_*j*3*i*_, which represents the *i* th gene expression of the *j*_3_ th sample; samples typically include disease samples and control samples. Tensor ${\tilde{x}}_{{j}_{1}{j}_{2}{j}_{3}i}$ was generated as a ‘mathematical product’ of *x*_*j*1*j*2*i*_ and *x*_*j*3*i*_. Then, tensor ${\tilde{x}}_{{j}_{1}{j}_{2}{j}_{3}i}$ is decomposed, and singular value matrix of compounds ${u}_{{\ell }_{1}{j}_{1}}$, singular value matrix of time points ${u}_{{\ell }_{2}{j}_{2}}$, sample singular value matrix ${u}_{{\ell }_{3}{j}_{3}}$, and gene singular value matrix ${u}_{{\ell }_{4}i}$ are obtained. Among them, I selected the combinations of ${\ell }_{k}\mathrm{,1}\le k\le 4$, which are simultaneously associated with all of the following: i) core tensor $G({\ell }_{1},{\ell }_{2},{\ell }_{3},{\ell }_{4})$ with a large enough absolute value, ii) a singular value vector of time points, ${u}_{{\ell }_{2}{j}_{2}}$, whose value significantly varies with time, and iii) sample singular value vector ${u}_{{\ell }_{3}{j}_{3}}$. These parameters are different between a disease (red filled circles) and control samples (cyan filled circles). Finally, using gene singular value vector ${u}_{{\ell }_{4}i}$ and compound singular value vector ${u}_{{\ell }_{1}{j}_{1}}$, compounds (filled pink circles) and genes (filled light-green circles) associated with $G({\ell }_{1},{\ell }_{2},{\ell }_{3},{\ell }_{4})$ s with large enough absolute values are selected. Next, if the selected genes are coincident with the genes associated with a significant alteration when gene *X* is knocked out (or overexpressed), then the compounds are assumed to target gene *X*.

The next task is to identify singular value vectors of genes and drugs to be used for identification of genes and drugs. This is because I could find combinations of singular value vectors of time points and samples that have the required properties, i.e. an association with time dependence and a difference between diseased and normal samples, as suggested in Fig. 4. These selection procedures are based on the association with core tensors, $G({\ell }_{1},\ldots ,{\ell }_{m})$, that have greater absolute values and are associated with sample and time point singular value vectors identified above. At the moment, we do not have any specific criteria for how to handle large $G({\ell }_{1},\ldots ,{\ell }_{m})$ s. After analysing the top 20 $G({\ell }_{1},\ldots ,{\ell }_{m})$ s with larger absolute values, I realised the following. Gene singular value vectors associated with the top 20 $G({\ell }_{1},\ldots ,{\ell }_{m})$ s generally vary from one $G({\ell }_{1},\ldots ,{\ell }_{m})$ to another $G({\ell }_{1},\ldots ,{\ell }_{m})$. In other words, each $G({\ell }_{1},\ldots ,{\ell }_{m})$ was associated with distinct singular value vectors of genes. Thus, tentatively, I decided to use the top 10 $G({\ell }_{1},\ldots ,{\ell }_{m})$ s and gene singular value vectors associated with them. Because the selected genes based on these singular value vectors are not assumed to directly encode drug target proteins but are simply used for identifying genes encoding drug target proteins (see the region within the rectangle with rounded corners in Fig. 4), I presumed that these specific criteria do not drastically affect identification of genes encoding drug target proteins. In contrast, compound singular value vectors are more restricted. In other words, $G({\ell }_{1},\ldots ,{\ell }_{m})$ s are associated with common compound singular value vectors. Thus, the selection of compound singular value vectors used for drug selection is more robust.

As for heart failure, $G({\ell }_{1}{\ell }_{2}{\ell }_{3}{\ell }_{4}),{l}_{2}=\mathrm{2,}\,2\le {l}_{3}\le 3$ were studied. Because absolute values of $G({\ell }_{1},\ldots ,{\ell }_{4})$ s gradually decrease as the rank of $G({\ell }_{1},\ldots ,{\ell }_{4})$ s decreases, there are no clear threshold values of $G({\ell }_{1},\ldots ,{\ell }_{4})$ above which $G({\ell }_{1},\ldots ,{\ell }_{4})$ s are used. Therefore, I selected the top 10 singular value vectors of genes, ${\ell }_{4}=\mathrm{21,}\,\mathrm{25,}\,\mathrm{27,}$ $\mathrm{28,}\,\mathrm{33,}\,\mathrm{36,}\,\mathrm{37,}\,\mathrm{38,}\,\mathrm{41,}\,42$, which are used for identification of genes with altered expression. Genes encoding drug target proteins are later identified by the enrichment analysis of these genes in terms of the genes associated with altered gene expression when genes encoding drug target proteins are perturbed. Nevertheless, because only the second singular value vector of compounds is associated with the top 20 $G({\ell }_{1},\ldots ,{\ell }_{4})$ s, I decided to employ the second singular value vectors of compounds (${\ell }_{1}=2$) for use in drug identification later.

### PTSD

In gene expression profiles of the rat brain treated with 22 drugs, I selected four time points (1/4, 1, 3, and 5 days after treatment); this is because there were substantially smaller numbers of compounds tested for other time points. Rat hippocampus and amygdala gene expression profiles of 15 males and 15 females are composed of five control samples, five minimal behavioural response samples, and five extreme behavioural response samples, respectively. In these profiles, 7501 genes sharing gene symbols between the two experiments were considered. Then, the generated tensor is

2 $${\tilde{x}}_{{j}_{1}{j}_{2}{j}_{3}{j}_{4}{j}_{5}i}={x}_{{j}_{1}{j}_{2}i}\cdot {x}_{{j}_{3}{j}_{4}i}\cdot {x}_{{j}_{3}{j}_{5}i},$$

where ${\tilde{x}}_{{j}_{1}{j}_{2}{j}_{3}{j}_{4}{j}_{5}i}\in {{\mathbb{R}}}^{22\times 4\times 2\times 15\times 7501}$, which represents the mathematical product of gene expression of the *i* th gene of a brain treated with the *j*_1_ th compound at the *j*_2_ th time point after the drug treatment, ${x}_{{j}_{1}{j}_{2}i}\in {{\mathbb{R}}}^{22\times 4\times 7501}$, gene expression of the *j*_3_ th brain region (hippocampus: *j*_3_ = 1, and amygdala: *j*_3_ = 2) of the *j*_4_ th female sample and *j*_5_ th male sample (control samples: 1 ≤ *j*_4_, *j*_5_ ≤ 5, minimal behavioural response samples: 6 ≤ *j*_4_, *j*_5_ ≤ 10, and extreme behavioural response samples: 11 ≤ *j*_4_, *j*_5_ ≤ 15), ${x}_{{j}_{3}{j}_{4}i}\in {{\mathbb{R}}}^{2\times 15\times 7501}$ and ${x}_{{j}_{3}{j}_{5}i}\in {{\mathbb{R}}}^{2\times 15\times 7501}$, respectively. HOSVD was applied to ${\tilde{x}}_{{j}_{1}{j}_{2}{j}_{3}{j}_{4}{j}_{5}i}$, and then core tensor $G({\ell }_{1}{\ell }_{2}{\ell }_{3}{\ell }_{4}{\ell }_{5}{\ell }_{6})\in {{\mathbb{R}}}^{22\times 4\times 2\times 15\times 7501}$, compound singular value matrix ${u}_{{\ell }_{1}{j}_{1}}\in {{\mathbb{R}}}^{22\times 22}$, time point singular value matrix ${u}_{{\ell }_{2}{j}_{2}}\in {{\mathbb{R}}}^{4\times 4}$, singular value matrix of a rat brain region ${u}_{{\ell }_{3},{j}_{3}}\in {{\mathbb{R}}}^{2\times 2}$, rat sample singular value matrices ${u}_{{\ell }_{4}{j}_{4}}\in {{\mathbb{R}}}^{15\times 15}$ and ${u}_{{\ell }_{5}{j}_{5}}\in {{\mathbb{R}}}^{15\times 15}$, and gene singular value matrix ${u}_{{\ell }_{6}i}\in {{\mathbb{R}}}^{7501\times 7501}$ were obtained.

Prior to selection of genes and compounds, we need to know which singular value vector of time points, ${u}_{{\ell }_{2}{j}_{2}}$, represents time dependence (Fig. 2). Then, I decided to use the second singular value vectors (${\ell }_{2}=2$) for PTSD.

In relation to the rat model of PTSD, the set of samples is composed of 30 female and male samples, and each of these groups represents 15 hippocampus samples and 15 amygdala samples. Fifteen rats were subdivided into five controls, five minimal-behavioural-response samples, and five extreme-behavioural-response ones. Figure 3B shows the comparisons of sample singular value vectors ${u}_{{\ell }_{k}{j}_{k}}(k=\mathrm{4,}\,\mathrm{5)}$ between 10 male and female controls (1 ≤ *j*_*k*_ ≤ 5), 10 minimal-behavioural-response males and females (6 ≤ *j*_*k*_ ≤ 10), and 10 extreme-behavioural-response males and females (11 ≤ *j*_*k*_ ≤ 15), respectively. Next, the third singular value vector of samples, ${u}_{{\ell }_{k}\mathrm{=3,}{j}_{k}},k=\mathrm{4,}\,5$, turned out to show a significant difference between controls and others. Thus, I decided to employ ${u}_{{\ell }_{k}\mathrm{=3,}{j}_{k}},k=\mathrm{4,}\,5$ to determine which singular value vectors of genes and drugs should be used for selection of genes and drugs in the analysis below. Although the *P*-value associated with ${u}_{{\ell }_{k}\mathrm{=3,}{j}_{k}},k=\mathrm{4,}\,5$ is relatively greater (weaker significance), this drawback is caused by the smaller number of samples. In actuality, the BH criterion correction does not make the *P*-value non-significant.

Regarding the PTSD rat model, $G({\ell }_{1}{\ell }_{2}{\ell }_{3}{\ell }_{4}{\ell }_{5}{\ell }_{6}),{\ell }_{2}=\,\mathrm{2,}{\ell }_{4}={\ell }_{5}=\,3$ were investigated. Given that absolute values of $G({\ell }_{1},\ldots ,{\ell }_{6})$ s again gradually decrease as the rank of $G({\ell }_{1},\ldots ,{\ell }_{6})$ s diminishes, there are no clear threshold values of $G({\ell }_{1},\ldots ,{\ell }_{6})$ above which $G({\ell }_{1},\ldots ,{\ell }_{6})$ s are employed. Therefore, I decided to select the top 10 singular value vectors of genes, ${\ell }_{6}=\mathrm{75,77,81,83,84,85,88,89,90,102}$, which are used for identification of genes with altered expression. Genes encoding drug target proteins are later inferred by the enrichment analysis of these genes in terms of the genes associated with altered gene expression when genes encoding drug target protein are perturbed. Because only the second singular value vector of compounds (${\ell }_{1}=2$) is associated with the top 20 $G({\ell }_{1},\ldots ,{\ell }_{6})$ s, I decided to use the second singular value vectors of compounds for drug identification later. Nonetheless, ${\ell }_{3}=1$ for all the 10 top-ranked $G({\ell }_{1},\ldots ,{\ell }_{6})$.

### ALL

From gene expression profiles of the rat bone narrow treated with 77 drugs, I selected four time points (1/4, 1, 3, and 5 days after treatment); this is because there were substantially smaller numbers of compounds tested for other time points than for these four. On the other hand, gene expression profiles of bone narrow in human (child) ALL represents 74 ALL patients at 0, 8, 15, and 33 days after a remission induction therapy. In these profiles, 2597 genes sharing gene symbols between rats and humans were considered. Then, the generated tensor is

3 $${\tilde{x}}_{{j}_{1}{j}_{2}{j}_{3}{j}_{4}i}={x}_{{j}_{1}{j}_{2}i}\cdot {x}_{{j}_{3}{j}_{4}i}$$

where ${\tilde{x}}_{{j}_{1}{j}_{2}{j}_{3}{j}_{4}i}\in {{\mathbb{R}}}^{77\times 4\times 4\times 74\times 2597}$, which represents the mathematical product of gene expression of the *i* th gene of rat bone narrow treated with the *j*_1_ th compound at the *j*_2_ th time point after the drug treatment, ${x}_{{j}_{1}{j}_{2}i}\in {{\mathbb{R}}}^{77\times 4\times 2597}$ and ALL bone narrow gene expression on the *j*_3_ th day after the remission induction therapy of the *j*_4_ th sample, ${x}_{{j}_{3}{j}_{4}i}\in {{\mathbb{R}}}^{4\times 74\times 2597}$, respectively. HOSVD was applied to ${\tilde{x}}_{{j}_{1}{j}_{2}{j}_{3}{j}_{4}i}$, and then core tensor $G({\ell }_{1}{\ell }_{2}{\ell }_{3}{\ell }_{4}{\ell }_{5})\in {{\mathbb{R}}}^{77\times 4\times 4\times 74\times 2597}$, compound singular value matrix ${u}_{{\ell }_{1}{j}_{1}}\in {{\mathbb{R}}}^{77\times 77}$, time point singular value matrix ${u}_{{\ell }_{2}{j}_{2}}\in {{\mathbb{R}}}^{4\times 4}$, a singular value matrix of ALL days, ${u}_{{\ell }_{3},{j}_{3}}\in {{\mathbb{R}}}^{4\times 4}$, a singular value matrix of ALL patients, ${u}_{{\ell }_{4}{j}_{4}}\in {{\mathbb{R}}}^{74\times 74}$, and gene singular value matrix ${u}_{{\ell }_{5}i}\in {{\mathbb{R}}}^{2597\times 2597}$ were obtained.

Prior to selection of genes and compounds, we need to know which singular value vector of time points, ${u}_{{\ell }_{2}{j}_{2}}$, represents time dependence (Fig. 2). After that, I decided to use the third vector (${\ell }_{2}=3$) for ALL because this vector has the strongest correlations with days.

Regarding ALL, in contrast to the other diseases, the given samples represent not diseases and controls but rather time progression of gene expression after therapy. Then, because the fourth singular value vector of time points (Fig. 3C), ${u}_{{\ell }_{3}\mathrm{=4,}{j}_{3}}$, has the strongest correlation with time points, I decided to use it for the further analysis in the text below. Although the first one, ${u}_{{\ell }_{3}\mathrm{=1,}{j}_{3}}$, also shows a moderate correlation, because the gradient is much smaller, I did not select the first vector.

As for ALL, $G({\ell }_{1}{\ell }_{2}{\ell }_{3}{\ell }_{4}{\ell }_{5}),{\ell }_{2}=3,\,{\ell }_{3}=4$ were studied. Although $G({\ell }_{1},\ldots ,{\ell }_{5})$ with the largest absolute value is exceptionally big, lower-ranked $G({\ell }_{1},\ldots ,{\ell }_{5})$ s are not small enough to be ignored. Therefore, I decided to employ the top 10 $G({\ell }_{1},\ldots ,{\ell }_{5})$ s again. In contrast to the above two cases (heart failure and rat model of PTSD), both gene and compound singular value vectors associated with the top 20 $G({\ell }_{1},\ldots ,{\ell }_{5})$ s vary depending on $G({\ell }_{1},\ldots ,{\ell }_{5})$ s. Thus, I decided to select gene singular value vectors ${\ell }_{5}=\mathrm{1,2,3,5}$ and compound singular value vectors ${\ell }_{1}=\mathrm{2,}\,\mathrm{3,}\,\mathrm{5,}\,\mathrm{6,}\,\mathrm{9,}\,10$, which are used for identification of genes with altered expression. Genes encoding drug target proteins are later inferred by the enrichment analysis of these genes in terms of the genes associated with altered gene expression when genes encoding drug target proteins are perturbed. The reason why the number of vectors selected is less than 10, which is the number of $G({\ell }_{1},\ldots ,{\ell }_{5})$ s being analysed, is that each singular value vector is associated with more than one $G({\ell }_{1},\ldots ,{\ell }_{5})$.

### Diabetes

In gene expression profiles of the rat kidney treated with 253 drugs, I selected four time points (1/4, 1, 3, and 5 days after treatment); this is because there were substantially smaller numbers of compounds tested for other time points than for these four. Kidney gene expression of 50 controls (25 whole kidneys, 12 tubuli, and 13 glomeruli) and 19 diabetic kidney samples were considered. In these data, 3489 genes sharing gene symbols between rats and humans were analysed. Then the generated tensor is

4 $${\tilde{x}}_{{j}_{1}{j}_{2}{j}_{3}i}={x}_{{j}_{1}{j}_{2}i}\cdot {x}_{{j}_{3}i}$$

where ${\tilde{x}}_{{j}_{1}{j}_{2}{j}_{3}i}\in {{\mathbb{R}}}^{253\times 4\times 69\times 3489}$, which represents the products of gene expression of the *i* th gene of the rat kidney treated with the *j*_1_ th compound at the *j*_2_ th time point after the drug treatment, ${x}_{{j}_{1}{j}_{2}i}\in {{\mathbb{R}}}^{253\times 4\times 3489}$ and gene expression of the *j*_3_ th human kidney sample, ${x}_{{j}_{3}i}\in {{\mathbb{R}}}^{69\times 3489}$, respectively. HOSVD was applied to ${\tilde{x}}_{{j}_{1}{j}_{2}{j}_{3}i}$, and then core tensor $G({\ell }_{1}{\ell }_{2}{\ell }_{3}{\ell }_{4})\in {{\mathbb{R}}}^{253\times 4\times 69\times 3489}$, compound singular value matrix ${u}_{{\ell }_{1}{j}_{1}}\in {{\mathbb{R}}}^{253\times 253}$, time point singular value matrix ${u}_{{\ell }_{2}{j}_{2}}\in {{\mathbb{R}}}^{4\times 4}$, human sample singular value matrix ${u}_{{\ell }_{3}{j}_{3}}\in {{\mathbb{R}}}^{69\times 69}$, and gene singular value matrix ${u}_{{\ell }_{4}i}\in {{\mathbb{R}}}^{3489\times 3489}$ were obtained.

Prior to selection of genes and compounds, we need to know which singular value vector of time points, ${u}_{{\ell }_{2}{j}_{2}}$, represents time dependence (Fig. 2). Then, I decided to use the second singular value vectors (${\ell }_{2}=2$) for diabetes.

As for diabetes, the first four singular value vectors of samples, ${u}_{{\ell }_{3}{j}_{3}}\mathrm{,1}\le {\ell }_{3}\le 4$, show a significant difference among four classes (Fig. 3D), which are composed of three healthy tissue classes and one diseased kidney class. Nonetheless, because the first and fourth vectors have greater significance, I decided to employ these two.

Regarding diabetes, $G({\ell }_{1}{\ell }_{2}{\ell }_{3}{\ell }_{4}),{\ell }_{2}=2,{\ell }_{3}=\mathrm{1,4}$ were investigated. Top two $G({\ell }_{1},\ldots ,{\ell }_{4})$ s with larger absolute values are outstanding. Accordingly, I selected the first and fourth singular value vectors of genes (${\ell }_{4}=\mathrm{1,4}$) and the second vector of compounds (${\ell }_{1}=2$) that are associated with the first two top-ranked $G({\ell }_{1},\ldots ,{\ell }_{4})$ s, which are used for identification of genes with altered expression. Genes encoding drug target proteins are later inferred by the enrichment analysis of these genes relative to the genes associated with altered gene expression when genes encoding drug target proteins are perturbed.

### Renal carcinoma

Rat kidney samples were also combined with human renal carcinoma samples, which represent 101 human patients (one tumour sample and one sample of adjacent non-tumourous renal tissue from each patient) because some correlation between diabetes and renal carcinoma has been reported^29^. If the present strategy works similarly for both diseases, then these results can strengthen its usefulness. Among genes included in rat kidney and renal carcinoma profiles, 4036 genes sharing gene symbols between rats and humans were considered. Accordingly, the generated tensor is

5 $${\tilde{x}}_{{j}_{1}{j}_{2}{j}_{3}i}={x}_{{j}_{1}{j}_{2}i}\cdot {x}_{{j}_{3}i}$$

where ${\tilde{x}}_{{j}_{1}{j}_{2}{j}_{3}i}\in {{\mathbb{R}}}^{253\times 4\times 202\times 4036}$, which represents the mathematical product of gene expression of the *i* th gene of the rat kidney treated with the *j*_1_ th compound at the *j*_2_ th time point after the drug treatment, ${x}_{{j}_{1}{j}_{2}i}\in {{\mathbb{R}}}^{253\times 4\times 4036}$, and gene expression of the *j*_3_ th human kidney sample, ${x}_{{j}_{3}i}\in {{\mathbb{R}}}^{202\times 4036}$, respectively. HOSVD was applied to ${\tilde{x}}_{{j}_{1}{j}_{2}{j}_{3}i}$, and then core tensor $G({\ell }_{1}{\ell }_{2}{\ell }_{3}{\ell }_{4})\in {{\mathbb{R}}}^{253\times 4\times 202\times 4036}$, compound singular value matrix ${u}_{{\ell }_{1}{j}_{1}}\in {{\mathbb{R}}}^{253\times 253}$, time point singular value matrix ${u}_{{\ell }_{2}{j}_{2}}\in {{\mathbb{R}}}^{4\times 4}$, human sample singular value matrix ${u}_{{\ell }_{3}{j}_{3}}\in {{\mathbb{R}}}^{202\times 202}$, and gene singular value matrix ${u}_{{\ell }_{4}i}\in {{\mathbb{R}}}^{4036\times 4036}$ were obtained.

Prior to selection of genes and compounds, we need to know which singular value vector of time points, ${u}_{{\ell }_{2}{j}_{2}}$, represents time dependence (Fig. 2). Then, I decided to use the second singular value vectors (${\ell }_{2}=2$) for renal carcinoma.

Regarding renal carcinoma, the samples represent 101 patients, from whom one tumorous and one healthy tissue sample were taken. In this case, the first several singular value vectors do not show a significant difference between healthy and tumorous tissue samples. Thus, I performed BH corrections on all the *P*-values attributed to 202 singular value vectors of samples, ${u}_{{\ell }_{3}{j}_{3}}\in {{\mathbb{R}}}^{202\times 202}$. Next, I found that five sample singular value vectors, ${\ell }_{3}=\mathrm{13,}\,\mathrm{15,}\,\mathrm{30,}\,\mathrm{33,}\,35$ (Fig. 3E), can be used to find singular value vectors of genes and drugs for identification of genes and drugs in the analysis below.

In relation to renal carcinoma, $G({\ell }_{1}{\ell }_{2}{\ell }_{3}{\ell }_{4}),{\ell }_{2}=\mathrm{2,}{\ell }_{3}=\mathrm{13,}\,\mathrm{15,}\,\mathrm{30,}\,\mathrm{33,}\,35$ were analysed. Although only the second singular value vector of compounds (${\ell }_{1}=2$) is associated with 20 top-ranked $G({\ell }_{1},\ldots ,{\ell }_{4})$ s with larger absolute values, various gene singular valued vectors are associated with 20 top-ranked $G({\ell }_{1},\ldots ,{\ell }_{4})$ s with larger absolute values. After that, gene singular value vectors ${\ell }_{4}=\mathrm{186,}\,\mathrm{215,}\,\mathrm{233,}\,\mathrm{244,}\,\mathrm{251,}\,\mathrm{269,}\,\mathrm{274,}\,\mathrm{309,}\,\mathrm{312,}\,318$ were selected, which are used for identification of genes with altered expression. Genes encoding drug target proteins are later inferred by the enrichment analysis of these genes regarding the genes associated with altered gene expression when genes encoding drug target proteins are perturbed.

### Cirrhosis

In gene expression profiles of the rat liver treated with 355 drugs, I selected four time points (1/4, 1, 3, and 5 days after treatment); this is because there were substantially smaller numbers of compounds tested for other time points. Human liver gene expression profiles of patients with cirrhosis are composed of 216 samples (60 correspond to a good prognosis, 101 to an intermediate prognosis, and 55 samples correspond to a poor prognosis). In these profiles, 3961 genes sharing gene symbols between rats and humans were considered. Then the generated tensor is

6 $${\tilde{x}}_{{j}_{1}{j}_{2}{j}_{3}i}={x}_{{j}_{1}{j}_{2}i}\cdot {x}_{{j}_{3}i}$$

where ${\mathop{x}\limits^{ \sim }}_{{j}_{1}{j}_{2}{j}_{3}i}\in {{\mathbb{R}}}^{355\times 4\times 216\times 3961}$, which represents the products of gene expression of the *i* th gene of the rat liver treated with the *j*_1_ th compound at the *j*_2_ th time point after the drug treatment, ${x}_{{j}_{1}{j}_{2}i}\in {{\mathbb{R}}}^{355\times 4\times 3961}$, and gene expression of the *j*_3_ th human liver sample, ${x}_{{j}_{3}i}\in {{\mathbb{R}}}^{216\times 3961}$, respectively. HOSVD was applied to ${\tilde{x}}_{{j}_{1}{j}_{2}{j}_{3}i}$, and then core tensor $G({\ell }_{1}{\ell }_{2}{\ell }_{3}{\ell }_{4})\in {{\mathbb{R}}}^{355\times 4\times 216\times 3961}$, compound singular value matrix ${u}_{{\ell }_{1}{j}_{1}}\in {{\mathbb{R}}}^{355\times 355}$, time point singular value matrix ${u}_{{\ell }_{2}{j}_{2}}\in {{\mathbb{R}}}^{4\times 4}$, human sample singular value matrix ${u}_{{\ell }_{3}{j}_{3}}\in {{\mathbb{R}}}^{216\times 216}$, and gene singular value matrix ${u}_{{\ell }_{4}i}\in {{\mathbb{R}}}^{3961\times 3961}$ were obtained.

Prior to selection of genes and compounds, we need to know which singular value vector of time points, ${u}_{{\ell }_{2}{j}_{2}}$, represents time dependence (Fig. 2). Then, I decided to use the second singular value vectors (${\ell }_{2}=2$) for cirrhosis.

As for cirrhosis, the second and sixth singular value vectors of samples, ${u}_{{\ell }_{3}\in \mathrm{(2,6),}{j}_{3}}$, show gradual and significant dependence on progression of diseases (Fig. 3F), from good to intermediate to poor prognosis. Accordingly, I decided to use these two. Because analysis of the sixth may be a bad approach, I performed BH criterion corrections on *P*-vales and confirmed that these two vectors are still significant.

As for cirrhosis, $G({\ell }_{1}{\ell }_{2}{\ell }_{3}{\ell }_{4}),{\ell }_{2}=\mathrm{2,}{\ell }_{3}=\mathrm{2,}\,6$ were investigated. Although only the second singular value vector of compounds (${\ell }_{1}\mathrm{=2}$) is again associated with 20 top-ranked $G({\ell }_{1},\ldots ,{\ell }_{4})$ s with larger absolute values, various gene singular value vectors are associated with 20 top-ranked $G({\ell }_{1},\ldots ,{\ell }_{4})$ s with larger absolute values. Next, gene singular value vectors $2\le {\ell }_{4}\le 10$ were selected, which are used for identification of genes with altered expression. Genes encoding drug target proteins are later inferred by the enrichment analysis of these genes regarding the genes associated with altered gene expression when genes encoding drug target proteins are perturbed.

### Identification of drugs and drug target proteins

By means of gene and compound singular value vectors ${u}_{{\ell }_{k},i}$ and ${u}_{{\ell }_{1},{j}_{1}}$, which were selected in the previous subsections and are listed in Table 1 and Fig. 1, drug candidates and genes encoding drug target proteins were identified as suggested in Fig. 4

**Figure 5:**
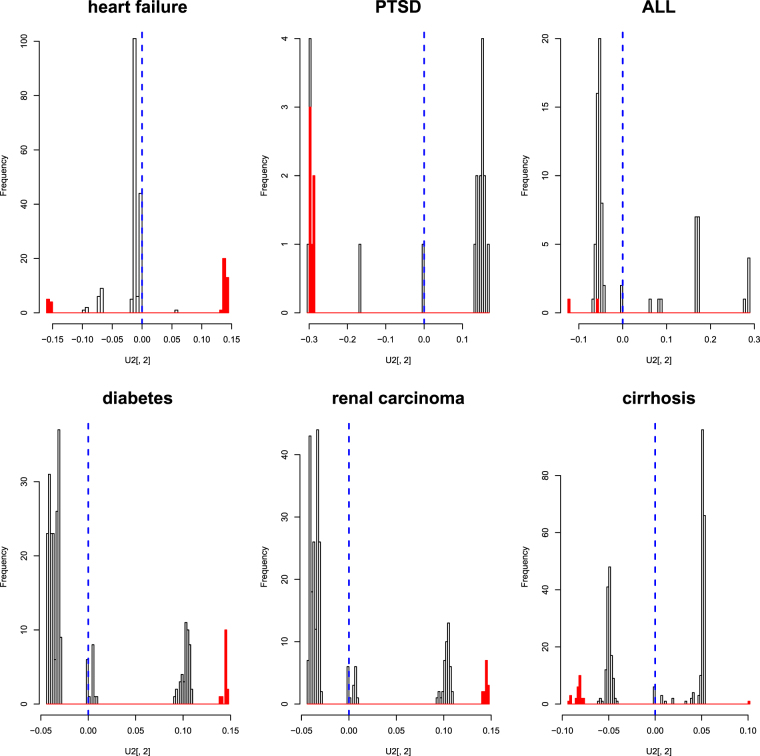
A histogram of the second singular value vectors of compounds. Red parts represent drugs selected as outliers, and vertical blue dashed lines are origins of the axes, by the distances from which the outliers were identified.

Firstly, I considered candidate drugs (for the full list, see Table S2). Except for ALL, the second singular value vector of compounds, ${u}_{{\ell }_{1}\mathrm{=2,}{j}_{1}}$, was identified. Thus, I decided to select outliers with it (Fig. 5). In the previous applications where PCA was used for identification of genes, I employed *P*-values attributed to each gene, assuming a *χ* ^2^ distribution. Unfortunately, this strategy could not be applied to the identification of candidate drugs. When trying to identify critical genes, I also observed gene expression of other, non-critical genes. Consequently, I could identify critical genes using comparisons with them. As for the drugs, however, drugs that are unlikely to affect the rat tissues being considered were not tested. Thus, in a sense, all the analysed drugs are seemingly critical. Therefore, I decided to identify drugs belonging to the cluster farthest from the origin. Because the definition of a cluster may be arbitrary, the selection of genes may not be unique (more detailed criteria and the list of selected drugs are presented in supplementary materials, Text S1).

Regarding identification of genes encoding drug target proteins, I utilised the following strategy as suggested in Fig. 4. Firstly, genes were selected using *P*-values computed from gene singular value vectors listed in Table 1 (*P*-values were calculated assuming *χ* ^2^ distributions for gene singular value vectors; genes associated with *P*-values adjusted by the BH criterion and less than 0.01 were selected; for the full list, see Table S2). Then, these genes were uploaded to Enrichr^30^ to identify genes encoding drug target proteins via ‘Single Gene Perturbations from GEO up’ and ‘Single Gene Perturbations from GEO down’ categories. After that, genes associated with adjusted *P*-values less than 0.01 were selected as genes encoding drug target proteins (Supporting Data S1).

### Biological evaluation of the selected compounds and genes

At first, it was tested whether known drug target proteins were enriched among those identified by the present strategy. To obtain the list of known drug target proteins, I used DINIES^31^. Although it can also infer unknown interactions between drugs and proteins, by uploading a single drug using a DINIES search with parameters ‘chemogenomic approach’ and ‘with learning on all DBs’, I obtained the list of target proteins of individual drugs (Supporting Data S2). Next, target genes were collected for individual drugs, and the collected sets of target genes were generated and used for further analysis in the text below.

To evaluate significant overlaps between the set of target genes predicted by DINIES and those with gene expression profiles, I carried out Fisher’s exact test and the uncorrected *χ* ^2^ test (Table 2). After that, I found that most of cases (10 out of 12 tables) are associated with significant *P*-values (Δ*G*

**Table 2:** Fisher’s exact test (*P*_*F*_) and the uncorrected *χ* ^2^ test (*P*_*χ*_^2^) of known drug target proteins regarding the inference of the present study.

		Single Gene Perturbations from GEO up					Single Gene Perturbations from GEO down				
		F	T	*P*_*F*_	*P*_χ_^2^	RO	F	T	*P*_*F*_	*P*_*χ*_^2^	RO
heart failure	F	521	517	3.4 × 10^−4^	3.9 × 10^−4^	3.02	628	416	1.3 × 10^−3^	7.3 × 10^−4^	2.61
	T	13	39				19	33			
PTSD	F	500	560	3.8 × 10^−2^	3.1 × 10^−2^	2.67	532	529	6.1 × 10^−3^	4.5 × 10^−3^	3.81
	T	6	18				5	19			
ALL	F	979	89	2.7 × 10^−1^	3.0 × 10^−1^	2.19	1009	57	1.0 × 10^0^	—	—
	T	10	2				12	0			
diabetes	F	889	177	1.2 × 10^−2^	7.1 × 10^−3^	3.00	936	130	3.6 × 10^−4^	2.0 × 10^−5^	5.13
	T	15	9				14	10			
renal carcinoma	F	847	219	2.0 × 10^−2^	1.2 × 10^−2^	2.75	895	169	4.3 × 10^−2^	2.2 × 10^−2^	2.64
	T	14	10				16	8			
cirrhosis	F	572	219	1.1 × 10^−2^	8.1 × 10^−3^	2.91	595	169	1.6 × 10^−3^	1.1 × 10^−3^	3.81
	T	8	10				7	8			

Rows: known drug target proteins (DINIES). Columns: Inferred drug target proteins using ‘Single Gene Perturbations from GEO up’ or ‘Single Gene Perturbations from GEO down’. OR: odds ratio.

) for both tests. Odds ratios are also generally approximately equal to three, which is usually considered large enough. Therefore, the proposed strategy improves identification of drug target proteins three-fold over random selection; this enhancement is large enough for effectiveness. Thus, it is obvious that this strategy is successful.

One may wonder whether Table 2 supports the usefulness of the present strategy because, generally, most genes identified as drug target genes by the present strategy are false positives although the inference and previous knowledge are significantly related. In this case, however, the situation is a little bit different from usual cases. For example, proteins inferred in the present study but not supported by previous knowledge may simply reflect the lack of experiments. In this case, these apparent false positives may turn out to be a true positive after suitable experimental validation. Alternatively, proteins supported by previous knowledge but not by the present study (i.e. false negatives) may simply reflect the lack of perturbation experiments. Proteins not included in perturbation experiments cannot be listed as true positives in the present study. This means that more perturbation experiments and additional validation of protein–drug interactions should be performed to validate the present results more precisely.

**Figure 6:**
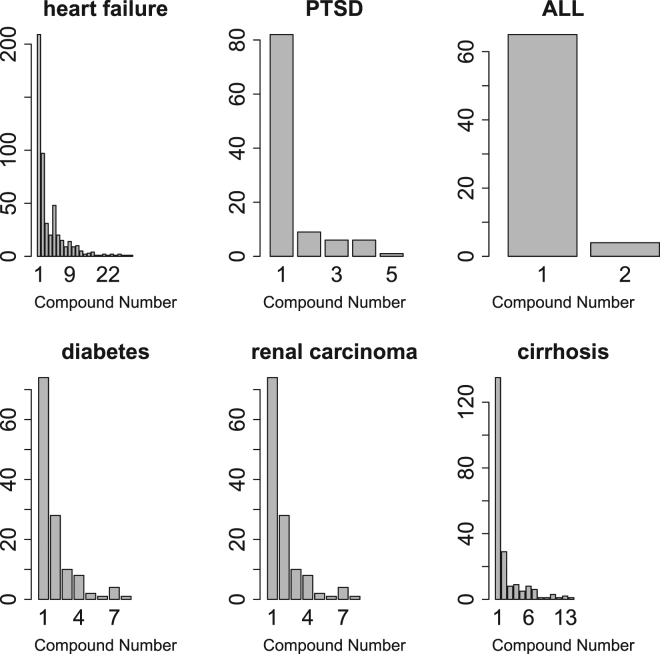
A histogram of the number of genes targeted by multiple compounds according to the results obtained after the identified drugs were uploaded to the DINIES server.

Readers may also wonder whether this kind of identification is useful because we are dealing with many-to-many correspondence not one-to-one correspondence. Nevertheless, target gene identification by DINIES was largely found to overlap among multiple compounds. As shown in Fig. 6, a substantial proportion of genes was targeted by more than a single compound, except for ALL where only two compounds were selected. In this sense, the proposed strategy successfully identifies a set of genes that share, to some extent, the compounds targeting these genes.

## Discussion

A question may arise whether the present study can be useful for real drug discovery experiments because there are more than a hundred genes listed for individual diseases (Table 2). To demonstrate the usefulness of the proposed strategy, in the text below, I specifically consider two genes, *CYPOR* and *HNF4A*, listed at the top for cirrhosis; CYPOR is top-ranked according to ‘Single Gene Perturbations from GEO up’, whereas HNF4A is top-ranked based on ‘Single Gene Perturbations from GEO down’. At present, there are no drugs directly targeting cirrhosis; thus, the strategy will hold some promise if we can propose some candidate drugs based on this analysis.

**Figure 7:**
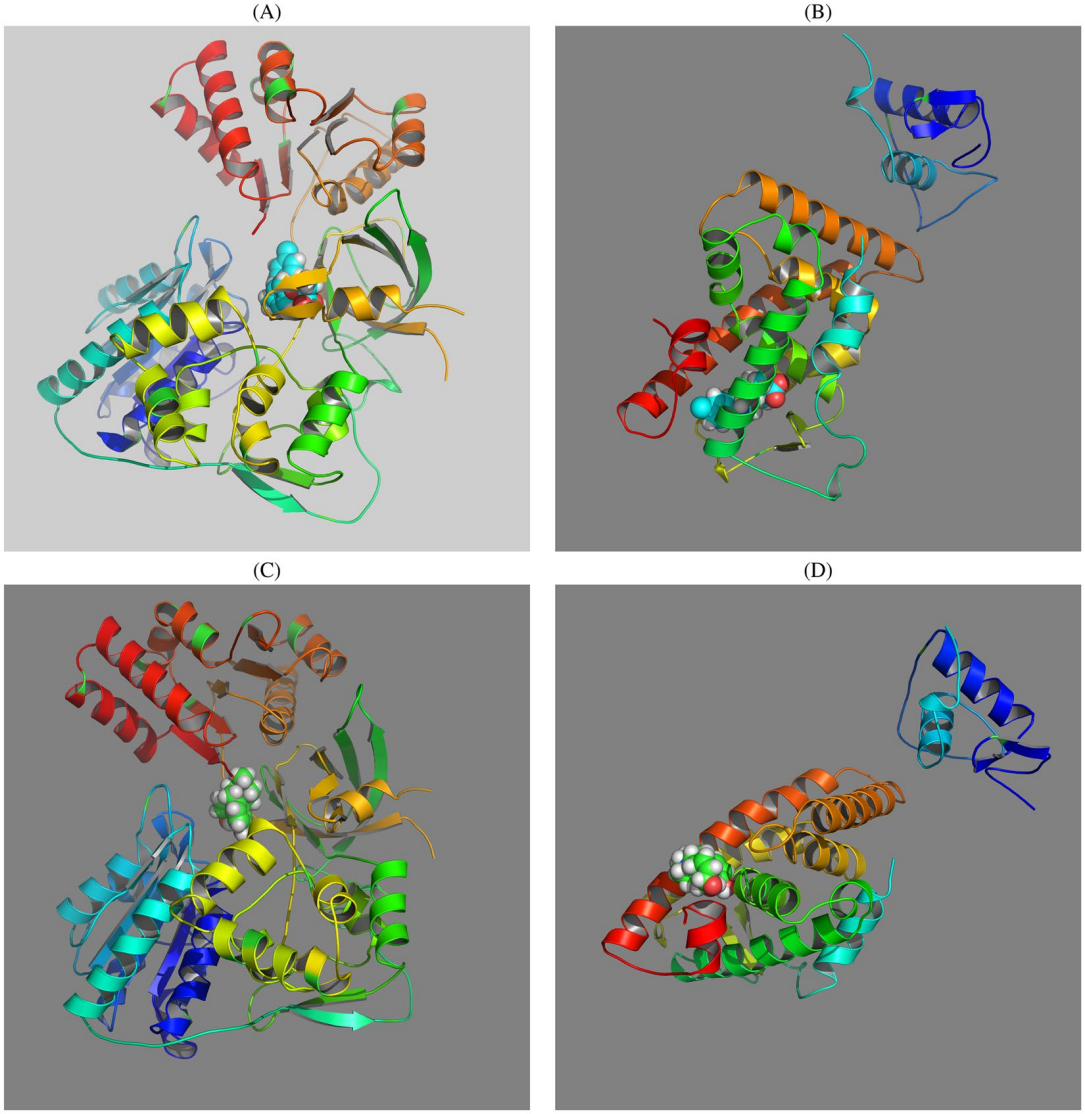
(**A**) Binding structure of bezafibrate toward CYPOR computed by SwissDock. (**B**) Binding structure of bezafibrate toward the ligand-binding domain of HNF4A computed by SwissDock. (**C**) Binding structure of morphine toward CYPOR computed by SwissDock. (**D**) Binding structure of morphine toward the ligand-binding domain of HNF4A computed by SwissDock.

Although the CYPOR protein has never been specifically studied in relation to cirrhosis, the activity of cytochrome P450 enzymes, to which CYPOR belongs, is regarded as important in this disease^32^. Accordingly, CYPOR may be an important therapeutic target in cirrhosis. Although no ligand compounds are known for CYPOR, bezafibrate was evaluated as a candidate ligand by *in silico* analysis (Fig. 7A). Its estimated binding energy, Δ*G*, is equal to 9.73 kcal, which corresponds to *K*_*i*_ = *exp*(−(Δ*G*)/(*RT*)) = 79 nM (*T* = 300 K), which is small enough to consider bezafibrate a useful ligand (*R* = 8.31 is the gas constant). Because bezafibrate itself is a known CYP inhibitor^33^, it is plausible that bezafibrate can be a drug for cirrhosis because of effects on CYPOR.

Although HNF4A has long been an orphan receptor, a ligand was identified recently^34^. Among the compounds identified by the present strategy, bezafibrate was estimated to bind to the ligand-binding domain of HNF4A with high affinity (Δ*G* = 9.44 kcal, which corresponds to 0.13 *μ*M at *T* = 300 K) by *in silico* analysis (Fig. 7B). Given that the structure of the ligand-binding domain is supposed to affect that of the DNA-binding domain^35^ (even can enhance the binding), and HNF4A is a transcription factor that can affect cirrhosis^36^, bezafibrate may also be a drug for cirrhosis from this point of view.

Although this is only one example and is limited to *in silico* analysis, if also considering that some researchers reported the efficiency of bezafibrate toward cirrhosis^37,38^, it indicates that the findings here can be a starting point for identification of new candidate drugs for diseases.

To evaluate the validity of this *in silico* analysis, I tried to assess binding of a possible negative control, morphine, which is unlikely to bind to either CYPOR or HNF4A (Fig. 7C and D). Morphine turned out to have relatively weak binding affinities (Δ*G* = 8.14 and 8.08 kcal, respectively, both of which correspond to 1.3 *μ*M). This result suggested that our strategy successfully identified drugs that can bind to target proteins with relatively strong binding affinity.

One may wonder whether only ten fold difference (0.13 *μ*M for bezafibrate vs 13 *μ*M for morphine) is significant enough or not. Nonetheless, these values should be evaluated not as the absolute values, but as the relative binding affinity towards proteins. In order to access the general binding affinity towards proteins, I accessed ChEMBL^39^ that stores vast number of experimentally validated compounds-protein interactions. Then I have found three records for morphine (CHEMBL409938), 0.32 nM with MOP, 11 nM with OPRD1, and 230 nM with OPRK1. On the other hand, I have found two records for bezafibrate (CHEMBL264374), 33 *μ*M for FABP2 and 44 *μ*M for FABP1. Thus, the best record of morphine in ChEMBL is more than 10^5^ times better than that of bezafibrate. Thus, our finding that bezafibrate is ten times better than morphine is definitely remarkable.

Finally, I would like to comment on the related studies. To my knowledge, there are no existing unsupervised methods comparable to the proposed one. The reason is as follows. To perform analyses similar to the present one, in a sense, multi-view data analyses^40^ are necessary, because two independent datasets, i.e. DrugMatrix and disease gene expression profiles, must be integrated. There are no existing unsupervised multi-view analytical methods by which more than one feature selection can be performed. For instance, Khan *et al*.^41^ proposed multi-tensor decomposition (thus, by definition, an unsupervised method) and applied it to drug discovery; they could identify only compounds and could not identify any drug target genes because by means of their methodology, only features shared across multiple views (in their case compounds) can be screened. Although Li^42^ proposed an integrated method that can identify drug–target protein interactions, the method is fully supervised because it cannot identify new drug–target protein interactions without any pre-knowledge.

Before closing the discussion, I would like to comment on the rationality of the present strategy. It is not obvious that applying TD to a mathematical product to obtain singular value vectors is guaranteed success because the relation between the mathematical product and the obtained singular value vectors is unclear. Nevertheless, the same problem had already existed when PCA was applied to gene expression. PCA is essentially matrix factorisation that approximates a matrix as a matrix product. This means that dimensionality of the resulting matrices can never be the same as that of the original gene expression matrix. As readers can see in eq. ((7)), the order of the products of singular value vectors is the same as the order of modes which can take any values. In this sense, dimensionality of the original matrix and that of the obtained matrix after application of TD to the original matrix can never match each other. Any kinds of matrix factorisation or TD simply represent assumptions that may become good approximations. If they work (in the present study, this word indicates that the resulting singular value vectors can be biologically interpretable), this means that the assumption is suitable. If not, it is not reliable. In this study, applying TD to a mathematical product clearly worked well, i.e. the obtained singular value vectors are biologically interpretable. Therefore, the proposed strategy is suitable for the present study and for the dataset being analysed, nothing more. I also would like to emphasize that the present strategy cannot fully replace the compounds or structure based approach that can screen huge number of compounds as many as millions. Until there are more gene expression profiles available, the present strategy must be regarded as only a supportive methodology toward these two main stream strategies.

## Methods

### Mathematics of TD

In this subsection, I briefly discuss what TD is and how I apply it to the problem at hand. Suppose an *m*-mode tensor, *X*, whose components are denoted as ${x}_{{j}_{1},\ldots ,{j}_{m-1}i}\in {{\mathbb{R}}}^{{N}_{1}\times {N}_{2}\times \cdots \times {N}_{m}}$, represents expression of the *i* th gene under the *j*_*k*_ (*k* = 1, …, *m* − 1, *j*_*k*_ = 1, …, *N*_*k*_) conditions, examples of which are diseases, patients, tissues, and time points. Then, TD is defined as

7 $${x}_{{j}_{1},\ldots ,{j}_{m-1}i}=\sum _{{\ell }_{1},\ldots ,{\ell }_{m}}^{{N}_{1},\ldots ,{N}_{m}}G({\ell }_{1},\ldots ,{\ell }_{m})\cdot {u}_{{\ell }_{m}i}\cdot \prod _{k\mathrm{=1}}^{m-1}{u}_{{\ell }_{k}{j}_{k}}$$

where $G({\ell }_{1},\ldots ,{\ell }_{m})\in {{\mathbb{R}}}^{{N}_{1}\times {N}_{2}\times \cdots \times {N}_{m}}$ is a core tensor and ${u}_{{\ell }_{m}i}\in {{\mathbb{R}}}^{{N}_{m}\times {N}_{m}}$ and ${u}_{{\ell }_{k}{j}_{k}}\in {{\mathbb{R}}}^{{N}_{k}\times {N}_{k}}$ are singular value matrices that are supposed to be orthogonal to each other, i.e.

8 $$\sum _{i\mathrm{=1}}^{{N}_{m}}{u}_{{\ell }_{m}i}{u}_{{\ell }_{m^{\prime} }i}={\delta }_{{\ell }_{m}{\ell }_{m^{\prime} }}\,{\rm{and}}\,\sum _{{j}_{k}\mathrm{=1}}^{{N}_{k}}{u}_{{\ell }_{k}{j}_{k}}{u}_{{\ell }_{k^{\prime} }{j}_{k}}={\delta }_{{\ell }_{k}{\ell }_{k^{\prime} }}$$

where ${\delta }_{{\ell }_{m}{\ell }_{m^{\prime} }}$ and ${\delta }_{{\ell }_{k}{\ell }_{k^{\prime} }}$ are Kronecker’s delta. Because the number of $G({\ell }_{1}\ldots {\ell }_{m})$ is assumed to be as large as that of *x*_*j1*…*jm − 1i*_, this is obviously an overcomplete problem; therefore, there are no unique solutions. To solve TD uniquely, I specifically employed the HOSVD algorithm^43^ that attempts to attain TD such that a smaller number of core tensors and singular value vectors can represent *x*_*j1*…*jm − 1i*_ as much as possible. All the tensors are standardised such that ∑_*i*_ *x*_*j1*,…,*jm − 1i*_ = 0, ∑_*i*_ *x*_*j1*,…,*jm − 1i*_^2^ = *N*_*m*_ before TD is performed in the present study.

The advantages over more popular TD – parallel factor analysis (PARAFAC)^43^ – are as follows. First of all, PARAFAC is NP-complete; in other words, there are no known algorithms that derive PARAFAC with polynomial time. Especially, in the present analysis, singular value vectors associated with a smaller contribution were often useful; ${\ell }_{k}$ is often greater than 100 (see Table 1). Thus, rapid convergence of computation is required, which is not achieved by PARAFAC. Secondly, the advantage of PARAFAC is that it provides one-to-one relations between singular value vectors having common ${\ell }_{k}$. By contrast, as shown in Table 1, the observed correspondence between singular value vectors is not one to one but often many to many, in which case, HOSVD was more suitable. For these two reasons, I decided to conduct HOSVD instead of more popular PARAFAC.

### Tensor generation for integrated analysis

Often, there is a set of gene expression profiles of human cell lines or model animals treated with various compounds at multiple dose densities. DrugMatrix^44^ and LINCS^45^ are good examples although the former comprises only temporal gene expression after treatment with various drugs. Nonetheless, it is not easy to infer a drug’s action on diseases by means of only these gene expression profiles. Some kind of integrated analysis with disease gene expression profiles is necessary, but it is not so straightforward. Candidate drugs should satisfy these conditions:

- Gene expression in these profiles must significantly decrease or increase with the increasing dose density of a compound.
- Gene expression alteration caused by drug treatment must be significantly coincident with that associated with disease progression.

How these two independent significance values can be evaluated is unclear. For example, we can have two sets of significant gene expression alterations of the *i* th gene, {Δ*x*_*i*_}, caused by drug treatment and those of the *i*′ th gene, {Δ*x*′_*i*′_}, during disease progression, respectively. Firstly, we need to test whether the two sets of genes significantly overlap. Next, when there is a significant overlap, we have to determine whether these two gene expression alteration profiles correlate significantly. Furthermore, because the analysis is usually conducted among multiple compounds, all the significance evaluation must be corrected based upon a multiple-comparison criterion. This is obviously a complicated and unpromising strategy.

On the other hand, if we can have gene expression profiles expressed via a tensor, *x* *j*_1_…j_m__−__1_i, where *j*_*k*_, *k* = 1, …, *m* − 1 corresponds to drug candidates, dose density, and disease progression, we can easily evaluate a candidate drug using TD: eq. ((7)). If there are ${u}_{{\ell }_{k}{j}_{k}}$ values that represent significant dependence on dose densities and disease progression, then genes’ and compounds’ singular value vectors – that share core tensor $G({\ell }_{1},\ldots ,{\ell }_{m})$ having larger absolute values with these ${u}_{{\ell }_{k}{j}_{k}}$ s – can be used for the selection of genes as well as compounds as follows.

Suppose $\{{\ell }_{k}\}$ is a set of indices of genes’ or compounds’ singular value vectors that are associated with significant dose density dependence as well as dependence on disease progression. Genes and compounds can be identified that are associated with significant singular value vector components. For this purpose, *P*-values are attributed to each *i* th gene and *j*_*k*_ th compound assuming a *χ* ^2^ distribution,

9 $${P}_{i}={P}_{{\chi }^{2}}[ > \sum _{\{{\ell }_{m}\}}{(\frac{{u}_{{\ell }_{m}i}}{{\sigma }_{{\ell }_{m}}})}^{2}]\,\,or\,\,{P}_{{j}_{k}}={P}_{{\chi }^{2}}[ > \sum _{\{{\ell }_{k}\}}{(\frac{{u}_{{\ell }_{k}{j}_{k}}}{{\sigma }_{{\ell }_{k}}})}^{2}]$$

where *P*_*χ*2_[>*x*] is the cumulative probability that the argument is greater than *x* assuming the *χ* ^2^ distribution, and ${\sigma }_{{\ell }_{m}}$ and ${\sigma }_{{\ell }_{k}}$ are standard deviations. After adjustment of *P*-values via the BH criterion^46^, genes and compounds that have significant *P*-values, i.e. less than 0.01, are selected as those contributing to the specified singular value vectors. Nonetheless, because such a tensor can be obtained only when drug treatment is performed on patients, this strategy is useless; if we can test drug efficiency directly on patients, then there is no need for *in silico* drug discovery. To overcome this obstacle, I replace *x* *j*_1_…j_m__−__1_i with a ‘mathematical product’; hereafter, this means that each component is defined as a product of two components, i.e.

10 $${\tilde{x}}_{{j}_{1}\ldots {j}_{m-1}i}={x}_{{j}_{1}\ldots {j}_{m^{\prime} }i}\cdot {x}_{{j}_{1}\ldots {j}_{m \textquotedbl }i},$$

where ${x}_{{j}_{1}\ldots {j}_{m^{\prime} }i}\in {{\mathbb{R}}}^{{N}_{1}\times \cdots \times {N}_{m^{\prime} }}$ is gene expression for the drug treatment of cell lines or model animals, whereas ${x}_{{j}_{1}\ldots {j}_{m \textquotedbl }i}\in {{\mathbb{R}}}^{{N}_{1}\times \cdots \times {N}_{m \textquotedbl }}$ is gene expression for the patients (*m* − 1 = *m*′ + *m*″). Because these two datasets can be obtained independently, we can test any kind of combinations of drug treatments and diseases even after all measurements were finalised. It is also obvious that this strategy retains the advantages of integrated analysis using PCA-based unsupervised FE because it still does not require assignment of any weights to each gene’s expression. On the other hand, it compensates the shortcoming of the integrated analysis of PCA-based unsupervised FE. Because this strategy identifies genes by considering expression of two genes in an integrated manner, there is no need to use common sets among multiple genes selected within an individual dataset as in the PCA based unsupervised FE.

### Explanation of the present strategy

Here is an accessible description of the present strategy (Fig. 4). It is obvious that the success of this seemingly complicated method heavily depends upon whether I can easily (or usually, in other words) obtain such preferable combinations of singular value vectors that have the properties illustrated in Fig. 4. Although this situation is unlikely to happen often, it seems to be possible in many cases as shown in the text above.

### Gene expression profiles

#### Heart failure

Gene expression profiles of the heart for drug treatments of rats were retrieved from DrugMatrix under the gene expression omnibus (GEO) ID GSE59905, whereas human gene expression for heart failure was retrieved from GEO ID 57345. For both datasets, expression files of genes GSE57345-GPL11532_series_matrix.txt.gz, GSE59905-GPL5426_series_matrix.txt.gz, and GSE59905-GPL5425_series_matrix.txt.gz were directly downloaded from the series matrix.

#### The rat model of PTSD

Gene expression profiles of the brain for drug treatments of rats were retrieved from DrugMatrix under GEO ID GSE59895, whereas amygdala and hippocampus gene expression for the rat model of PTSD was taken from GEO ID GSE60304. For both datasets, expression files of genes GSE60304_series_matrix.txt.gz, GSE59895-GPL5425_series_matrix.txt.gz, and GSE59895-GPL5426_series_matrix.txt.gz were directly downloaded from the series matrix.

#### ALL

Gene expression profiles of bone marrow for drug treatments of rats were retrieved from DrugMatrix under GEO ID GSE59894, and ALL human bone marrow gene expression was taken from GEO ID GSE67684. For both datasets, expression files of genes GSE67684-GPL570_series_matrix.txt.gz, GSE67684-GPL96_series_matrix.txt.gz, GSE59894-GPL5425_series_matrix.txt.gz, and GSE59894-GPL5426_series_matrix.txt.gz were directly downloaded from the series matrix.

#### Diabetes and renal cancer

Gene expression profiles of kidneys for drug treatments of rats were retrieved from DrugMatrix under GEO ID GSE59913, whereas gene expression for diabetic human kidneys and renal cancer was obtained from GEO ID GSE30122 and GSE40435, respectively. For these datasets, expression files of genes GSE30122_series_matrix.txt.gz, GSE40435_series_matrix.txt.gz, GSE59913-GPL5424_series_matrix.txt.gz, GSE59913-GPL5425_series_matrix.txt.gz, and GSE59913-GPL5426_series_matrix.txt.gz were directly downloaded from the series matrix.

#### Cirrhosis

Gene expression profiles of the liver for drug treatments of rats were retrieved from DrugMatrix under GEO ID GSE59923, whereas gene expression for cirrhosis of the human liver was obtained from GEO ID GSE15654. For both datasets, GSE15654_series_matrix.txt.gz, GSE59923-GPL5424_series_matrix.txt.gz, GSE59923-GPL5425_series_matrix.txt.gz, and GSE59923-GPL5426_series_matrix.txt.gz were directly downloaded from the series matrix.

### An enrichment analysis server

Gene symbols were uploaded to Enrichr^30^. Then, ‘Single Gene Perturbations from GEO up’ and ‘Single Gene Perturbations from GEO down’ categories were downloaded.

### Statistical analysis

All the statistical analyses were conducted in the R software. HOSVD was carried out using the hosvd function in the rTensor package.

### *In silico* evaluation of candidate drugs using SwissDock

SwissDock^47^ was used for the *in silico* evaluation of binding affinity of the identified drugs for a prospective protein. For CYPOR, Protein Data Bank^48^ (PDB) ID 1J9Z_A^49^ was used to retrieve protein structure, and for HNF4A, PDB ID 4IQR_A^50^ was used for this purpose. Compound structures provided by SwissDock when I searched by drug names, bezafibrate or morphine, were analysed. All the default options were chosen, and the binding structures associated with the smallest Δ*G* were selected.

## Electronic supplementary material

Text S1

Table S1

Table S2

Data S1

Data S2F

Data S2E

Data S2D

DataS2C

Data S2B

Data S2A
