# Supplementary material for: Identification of candidate drugs using tensor-decomposition-based unsupervised feature extraction in integrated analysis of gene expression between diseases and DrugMatrix datasets
Source: Sci Rep. 2017 Oct 23;7:13733. doi: 10.1038/s41598-017-13003-0 (PMC5653784; doi:10.1038/s41598-017-13003-0)
Supplement: Supplementary file 1 — Text S1 [file 41598_2017_13003_MOESM1_ESM.pdf]

Identification of candidate drugs using tensor-decomposition-based unsupervised feature extraction in integrated analysis of gene expression between diseases and DrugMatrix datasets

Y-h. Taguchi, Department of Physics, Chuo University, Tokyo 112-8551, Japan.

Detailed criteria of drug compound identification based upon compound singular value vectors

heart failure

Criterion

$|x_{\ell_1=2,j_1}| > 0.1$

number of drugs selected

43

PTSD

Criterion

$x_{\ell_1=1,j_1} < -0.15, x_{\ell_1=2,j_1} < -0.2$

number of drugs selected

6

ALL

Criterion

$$P_{j_1} = P_{\chi^2}\left[> \sum_{\ell_1 \in \{2,3,5,6,9,10\}} \left(\frac{x_{\ell_1,j_1}}{\sigma_{\ell_1}}\right)^2 \right]$$

j_1 associated with BH criterion adjusted P-values < 0.1.

number of drugs selected

2

diabetes/renal carcinoma

Criterion

$x_{\ell_1=2,j_1} > 0.13$

number of drugs selected

14

cirrhosis

Criterion

$|x_{\ell_1=2,j_1}| > 0.075$

number of drugs selected

27
